# Case Report: Immune checkpoint-inhibitor related myotoxicity monitoring using a comprehensive cardiothoracic MRI approach: insights from a clinical case

**DOI:** 10.3389/fcvm.2025.1527048

**Published:** 2025-02-14

**Authors:** Samia Boussouar, Etienne Charpentier, Baptiste Abbar, Jesus Gonzalez, Thomas Similowski, Mathieu Kerneis, Yves Allenbach, Marie Bretagne, Joe Elie Salem, Alban Redheuil

**Affiliations:** ^1^ Cardiovascular and Thoracic Imaging Unit, Hôpital Pitié-Salpêtrière, Assistance Publique-Hôpitaux de Paris (APHP), Paris, France; ^2^ Sorbonne Université, INSERM CIC-1901, AP.HP.Sorbonne, Department of Pharmacology, UNICO-GRECO Cardio-Oncology Program, Pitié-Salpêtrière Hospital, Paris, France; ^3^ Sorbonne Université, INSERM, UMRS1158 Neurophysiologie Respiratoire Expérimentale et Clinique, AP-HP, Groupe Hospitalier Universitaire APHP-Sorbonne Université, Hôpital Pitié-Salpêtrière, Département R3S (Respiration, Réanimation, Réhabilitation respiratoire, Sommeil), Paris, France; ^4^ INSERM U1166 Recherche sur les maladies Cardiovasculaires du Métabolisme et de la Nutrition, Hôpital Pitié-Salpêtrière, Assistance Publique-Hôpitaux de Paris (APHP), Paris, France; ^5^ Sorbonne Université, Assistance Publique - Hôpitaux de Paris, INSERM U974, Department of Internal Medicine and Clinical Immunology, Hôpital Pitié-Salpêtrière, Assistance Publique-Hôpitaux de Paris (APHP), Paris, France; ^6^ Sorbonne Université, INSERM, Institut Pierre Louis d'Epidémiologie et de Santé Publique (iPLESP), Assistance Publique-Hôpitaux de Paris (APHP), Pitié-Salpêtrière Hospital, Department of Medical Oncology, Institut Universitaire de Cancérologie, CLIP² Galilée, Paris, France; ^7^ Laboratoire d'Imagerie Biomédicale, Sorbonne Université, INSERM, CNRS, Institute of Cardiometabolism and Nutrition, Paris, France

**Keywords:** MRI, immune check inhibitor (ICI), diaphragm, thoracic, cardiac, myositis, diagnosis

## Abstract

Recent advances in immunotherapy have significantly improved outcomes for cancer patients. However, therapies such as immune checkpoint inhibitors (ICIs) can lead to immune-related adverse events, including potentially fatal ICI-myocarditis. The diagnosis of ICI-myocarditis is complex, and cardiac MRI plays a crucial role in identifying this condition. This report presents a novel finding of elevated muscular T1 and T2 relaxation parameters using standard CMR sequences, correlating with the clinical progression of ICI-myotoxicity affecting not only the heart but also the skeletal thoracic muscles and the diaphragm. Identifying diaphragm involvement in these patients is particularly important, as it may result in respiratory failure. MRI has shown significant potential in assessing diaphragmatic function, as well as detecting tissue damage and edema, while also monitoring their evolution over time. This comprehensive, imaging-based approach could guide treatment decisions, including the use of corticosteroids and immunosuppressive therapies. Combining functional and non-invasive tissue assessments in a single MRI examination may enhance early diagnosis and improve the management of patients undergoing ICI therapy.

## 1 Introduction

A 79-year-old male patient was admitted to the intensive care unit due to rapidly progressive dyspnea, without associated chest pain or hyperthermia. His clinical course was further complicated by rapid paroxysmal atrial fibrillation and sinus node dysfunction. His medical history was significant for a recent diagnosis, one month earlier, of metastatic lung adenocarcinoma with a PD-L1 expression of 1% and no detected EGFR, ALK, KRAS, BRAF, or HER2 mutations. Initial therapy included a combination regimen of Carboplatin, Pemetrexed, and Pembrolizumab, an immune checkpoint inhibitor (ICI).

## 2 Case description

Laboratory investigations revealed markedly elevated circulating levels of cardiac troponin-T (1,816 ng/L; normal <14 ng/L) and creatine kinase (CK, 1,369 IU/L; normal <160 IU/L). Transthoracic echocardiography demonstrated a left ventricular ejection fraction of 50% with elevated filling pressures (E/e’ >15 mmHg). Coronary angiography was normal, cardiac magnetic resonance imaging (cMRI) was suggestive of myocarditis, and myocardial biopsies showed non-specific fibrosis. A muscular biopsy confirmed features consistent with ICI-induced myositis, and electromyography findings supported a myogenic syndrome. Shortly after admission, the patient developed severe acute hypercapnic respiratory failure (PaCO₂ = 78 mmHg, pH = 7.28), necessitating mechanical ventilation. Clinical suspicion of diaphragmatic paralysis arose due to the presence of abdominal paradoxical respiration. This diagnosis was confirmed by the absence of diaphragmatic motion on ultrasound and the lack of inspiratory pressure or abdominal movement in response to bilateral magnetic phrenic nerve stimulation (1). Overall, this patient presented with diffuse myotoxicity involving the myocardium and skeletal muscles including the respiratory muscles.

More specifically, cMRI (1.5 Tesla MRI Aera Siemens, Germany) revealed a non-dilated, non-hypertrophied LV with mildly reduced ejection fraction (51%) and no regional wall motion abnormalities. There was no late gadolinium enhancement (LGE), but T2-mapping (T2pSSFP routine sequence) showed moderate and diffuse myocardial edema (T2 value = 56 ms compared to local reference values T2 = 48 ms +/−5 ms), and native T1 (985 ms, using the routine MOLLI sequence) was normal with a calculated extracellular volume of 29%. Mild pericardial and pleural effusions were present. Concomitant dynamic diaphragmatic MRI revealed an akinetic, thin diaphragm (maximum thickness: 4 mm) with associated edema of the diaphragmatic crura (T2 value = 66 ms compared to local reference values: T2 = 50 ms) and a markedly elevated ECV (88%) calculated using neighboring descending aorta blood pool values. These findings were consistent with diaphragmatic myositis. Edema of the psoas and scapularis muscles was also observed using quantitative T2 imaging, as described in studies by Huber et al. (2), and was compatible with skeletal muscle inflammation.

The patient was treated with a combination of immunosuppressants, including corticosteroids, ruxolitinib, and abatacept (3). This therapeutic approach has been previously reported as an effective strategy for managing fulminant ICI-related myocarditis. Corticosteroids were administered for their broad anti-inflammatory properties, while ruxolitinib and abatacept targeted specific immune pathways, offering a tailored treatment strategy to improve outcomes in high-risk patients (3, 4). Myocarditis resolved with normalization of troponin-T levels; however, persistent sinus node dysfunction necessitated permanent pacemaker implantation. Myositis showed gradual improvement, with the patient progressing from tetraplegia to the ability to ambulate with assistance. Concurrently, respiratory muscle function improved, allowing diurnal weaning from mechanical ventilation. The patient remained hospitalized throughout. This recovery was clinically evidenced by restored diaphragm movements on ultrasound and a progressive increase in maximum inspiratory pressure.

Three months after the initiation of immunosuppressive therapy, cMRI demonstrated a mild improvement in left ventricular ejection fraction (LVEF) to 55%, with complete resolution of myocardial edema (T2 = 47 ms) and a reduction in extracellular volume (ECV) to 24%. Diaphragmatic MRI showed the return of moderate bilateral diaphragmatic movements, accompanied by a significant decrease in diaphragmatic edema and ECV (64% vs. 88%) and complete resolution of edema in the psoas and scapular muscles. The follow-up MRI was conducted while the patient was off mechanical ventilation.

Pembrolizumab was permanently discontinued, and no alternative oncologic treatment was initiated due to the patient's frailty and the persistent sequelae of ICI-induced myotoxicity. Six months after the initial oncologic treatment, computed tomography revealed tumor progression, with new bone lesions and further enlargement of pulmonary and lymph node nodules.

Six weeks after receiving analgesic radiotherapy for bone metastases, the patient developed severe SARS-CoV-2 pneumonia, which was poorly tolerated. Dexamethasone and probabilistic antibiotic therapy were initiated; however, the clinical course was unfavorable. The patient succumbed to acute respiratory distress despite optimized supportive care.

### 2.1 A figure or table showcasing a timeline with relevant data from the episode of care

(This section would include a visual representation of the timeline, which is not provided in the text.)

### 2.2 Diagnostic assessment

Recent advances in immunotherapy have transformed the field of immuno-oncology, significantly improving survival outcomes for patients with various malignancies. However, the use of immune checkpoint inhibitors is limited by the occurrence of immune-related adverse events, which may also involve the cardiovascular system, including myocarditis. ICI-associated myocarditis is a rare but potentially life-threatening complication, with an incidence ranging from 0.1% to 1% and a case fatality rate of 30%–50% (4). This condition most commonly manifests within the first three months following the initiation of treatment.

The diagnosis of ICI-myocarditis is typically based on a combination of clinical symptoms, elevated troponin levels, cardiac magnetic resonance imaging (cMRI) findings (according to modified Lake Louise criteria), and/or endomyocardial biopsy (EMB). However, these diagnostic tools have limited individual sensitivity, making diagnosis challenging. Notably, normal ECG findings, unremarkable biomarkers, or preserved left ventricular ejection fraction (LVEF) do not exclude the presence of ICI-myocarditis (5–7). Strain imaging, derived from CMR or echocardiography, enables early detection of myocardial dysfunction, often preceding structural changes or alterations in LVEF. Studies underscore its value in identifying subclinical myocardial injury in ICI-treated patients (8, 9). Global longitudinal strain (GLS) in particular, serves as an independent risk factor for major adverse cardiovascular events, aids risk stratification and may identify early cardiotoxicity even with preserved LVEF, supporting timely interventions.A proposed framework suggests categorizing ICI-myocarditis as “definite,” “probable,” or “possible” based on the integration of multiple diagnostic modalities (10). A definite diagnosis requires the fulfillment of at least one of the following criteria: (1) histopathological evidence of myocarditis on EMB; (2) typical cMRI findings coupled with clinical syndromes, elevated cardiac biomarkers, or ECG abnormalities; or (3) the presence of new-onset wall motion abnormalities on echocardiography, in conjunction with clinical symptoms, elevated cardiac biomarkers, abnormal ECG findings, and negative coronary angiography (11, 12).

Classically, cMRI and endomyocardial biopsy are both considered relevant components of the diagnostic criteria. However, EMB is underutilized due to its invasive nature and the potential for rare but severe complications. Furthermore, myocardial biopsies are performed blindly, potentially at a distance from areas of active myocardial inflammation, resulting in high sampling bias with false-negative rate of EMB is roughly 40% (13). Subsequently, a negative EMB result should not rule out ICI- myocarditis but should always be interpreted within the clinical context, biological results, and cMRI findings (14).

ICI-related myocarditis currently lacks a gold standard diagnostic approach. Among non-invasive methods, cMRI is a well-established tool for diagnosing and prognosticating viral myocarditis. Although it has been extensively studied in the context of ICI-related cardiotoxicity, specific criteria for ICI-myocarditis remain lacking. cMRI offers detailed tissue characterization, detecting edema and fibrosis through T1 and T2 parametric mapping techniques, as well as late gadolinium enhancement imaging. Moreover, myocardial strain analysis, which can detect alterations in myocardial function with high sensitivity, appears to be associated with adverse outcomes in ICI-myocarditis, independent of LVEF (15, 16).

## 3 Discussion

The evaluation of diaphragm function relies on clinical examination, imaging, and physiological measures, with phrenic stimulation as the gold standard (1). Diaphragmatic MRI has been used to assess the continuity and mobility of the diaphragm, but tissue analysis of the diaphragm by mapping has not been previously described. Our study uniquely describes the use of MRI parametric T1 and T2 mapping on the crura of the diaphragm to evaluate the presence of edema and monitor its progression over time. Of significant relevance, the resorption of diaphragmatic edema over time in this patient was concomitant with measurable improvements in respiratory muscle function and clinical progress. However, we acknowledge that the normality cutoffs for T1 and T2 values are not yet defined, particularly in patients receiving ICI therapy. Moreover, measuring these parameters is challenging due to potential partial volume effects on small crura muscle volumes. Establishing a standardized methodology and expected value ranges for these parameters is essential before conducting studies eventually leading to guiding decisions on therapies in the future (5).

The severity of the disease is defined by the diffuse nature of myotoxicity affecting the heart, skeletal muscles, and, particularly, the diaphragm, potentially leading to fatal acute respiratory failure. In the present case, MRI findings were consistent with the clinical and biological presentation, involving the myocardium, peripheral muscles, and diaphragm. MRI is a precise, non-invasive modality that assesses morphological, functional, and tissue characteristics, enabling the evaluation of the heart, diaphragm, and peripheral thoracic muscles in a single examination. Although the EMB was negative in this case, it is prone to high sampling bias, highlighting the potential of cMRI in diagnosing ICI-myocarditis, even when EMB results are normal or inconclusive.

Comprehensive functional and parametric MRI of the heart, diaphragm, and peripheral muscles was successfully performed in this patient. MRI facilitated the assessment of the severity of affected organs and provided critical diagnostic and monitoring parameters that were reversible with the resolution of ICI-myotoxicity. This integrated, non-invasive diagnostic approach may enable more personalized management strategies for patients.

## 4 Patient perspective

This case provides critical insights that could advance the understanding, early recognition, and management of immune-related adverse events, particularly ICI-myocarditis as well as skeletal muscle and diaphragmatic myositis.

## Data Availability

The raw data supporting the conclusions of this article will be made available by the authors, without undue reservation.

## References

[1] Laveneziana P Albuquerque A Aliverti A Babb T Barreiro E Dres M ERS statement on respiratory muscle testing at rest and during exercise. Eur Respir J. (2019) 53(6):1801214. 10.1183/13993003.01214-2018 30956204

[2] Huber AT Lamy J Bravetti M Bouazizi K Bacoyannis T Roux C Comparison of MR T1 and T2 mapping parameters to characterize myocardial and skeletal muscle involvement in systemic idiopathic inflammatory myopathy (IIM). Eur Radiol. (2019) 29(10):5139–47. 10.1007/s00330-019-06054-6 30847587

[3] Nguyen LS Bretagne M Arrondeau J Zahr N Ederhy S Abbar B Reversal of immune-checkpoint inhibitor fulminant myocarditis using personalized-dose-adjusted abatacept and ruxolitinib: proof of concept. J Immunother Cancer. (2022) 10(4):e004699. 10.1136/jitc-2022-004699 35383117 PMC8984056

[4] Salem JE Manouchehri A Moey M Lebrun-Vignes B Bastarache L Pariente A Cardiovascular toxicities associated with immune checkpoint inhibitors: an observational, retrospective, pharmacovigilance study. Lancet Oncol. (2018) 19(12):1579–89. 10.1016/S1470-2045(18)30608-9 30442497 PMC6287923

[5] Lehmann LH Cautela J Palaskas N Baik AH Meijers WC Allenbach Y Clinical strategy for the diagnosis and treatment of immune checkpoint inhibitor-associated myocarditis: a narrative review. JAMA Cardiol. (2021) 6(11):1329–37. 10.1001/jamacardio.2021.2241 34232253

[6] Faron A Isaak A Mesropyan N Reinert M Schwab K Sirokay J Cardiac MRI depicts immune checkpoint inhibitor-induced myocarditis: a prospective study. Radiology. (2021) 301(3):602–9. 10.1148/radiol.2021210814 34581628

[7] von Kemp B Halvorsen S Nohria A. The new 2022 ESC guidelines on cardio-oncology and their impact on the acute cardiovascular care society. Eur Heart J Acute Cardiovasc Care. (2022) 11(11):844–9. 10.1093/ehjacc/zuac129 36205095

[8] Giusca S Korosoglou G Montenbruck M Geršak B Schwarz AK Esch S Multiparametric early detection and prediction of cardiotoxicity using myocardial strain, T1 and T2 mapping, and biochemical markers: a longitudinal cardiac resonance imaging study during 2 years of follow-up. Circ Cardiovasc Imaging. (2021) 14(6):e012459. 10.1161/CIRCIMAGING.121.012459 34126756 PMC8208092

[9] Zhao SH Yun H Chen CZ Chen YY Lin JY Zeng MS The prognostic value of global myocardium strain by CMR-feature tracking in immune checkpoint inhibitor-associated myocarditis. Eur Radiol. (2022) 32(11):7657–67. 10.1007/s00330-022-08844-x 35567603

[10] Bonaca MP Olenchock BA Salem JE Wiviott SD Ederhy S Cohen A Myocarditis in the setting of cancer therapeutics: proposed case definitions for emerging clinical syndromes in cardio-oncology. Circulation. (2019) 140(2):80–91. 10.1161/CIRCULATIONAHA.118.034497 31390169 PMC6779326

[11] Thavendiranathan P Zhang L Zafar A Drobni ZD Mahmood SS Cabral M Myocardial T1 and T2 mapping by magnetic resonance in patients with immune checkpoint inhibitor-associated myocarditis. J Am Coll Cardiol. (2021) 77(12):1503–16. 10.1016/j.jacc.2021.01.050 33766256 PMC8442989

[12] Tocchetti CG Farmakis D Koop Y Andres MS Couch LS Formisano L Cardiovascular toxicities of immune therapies for cancer - a scientific statement of the heart failure association (HFA) of the ESC and the ESC council of cardio-oncology. Eur J Heart Fail. (2024) 26(10):2055–76. 10.1002/ejhf.3340 39087551

[13] Ammirati E Buono A Moroni F Gigli L Power JR Ciabatti M State-of-the-art of endomyocardial biopsy on acute myocarditis and chronic inflammatory cardiomyopathy. Curr Cardiol Rep. (2022) 24(5):597–609. 10.1007/s11886-022-01680-x 35201561 PMC8866555

[14] Ederhy S Fenioux C Cholet C Rouvier P Redheuil A Cohen A Immune checkpoint inhibitor myocarditis with normal cardiac magnetic resonance imaging: importance of cardiac biopsy and early diagnosis. Can J Cardiol. (2021) 37(10):1654–6. 10.1016/j.cjca.2020.12.022 33373722

[15] Awadalla M Mahmood SS Groarke JD Hassan MZO Nohria A Rokicki A Global longitudinal strain and cardiac events in patients with immune checkpoint inhibitor-related myocarditis. J Am Coll Cardiol. (2020) 75(5):467–78. 10.1016/j.jacc.2019.11.049 32029128 PMC7067226

[16] Higgins AY Arbune A Soufer A Ragheb E Kwan JM Lamy J Left ventricular myocardial strain and tissue characterization by cardiac magnetic resonance imaging in immune checkpoint inhibitor associated cardiotoxicity. PLoS One. (2021) 16(2):e0246764. 10.1371/journal.pone.0246764 33606757 PMC7895343

